# Perioperative management of intracranial-extracranial communicating tumors with multidisciplinary combined surgery: a case series

**DOI:** 10.1186/s12871-025-03310-9

**Published:** 2025-08-27

**Authors:** Xiaoxiao Zhang, Liu Yang, Wenhao Wu, Ruquan Han, Haiyang Liu

**Affiliations:** 1 Department of Anesthesiology, Beijing Tiantan Hospital, Capital Medical University, 119 South 4th Ring West Road, Beijing, 100070 China; 2 Department of Neurosurgery, Beijing Tiantan Hospital, Capital Medical University, 119 South 4th Ring West Road, Beijing, 100070 China

**Keywords:** Anesthesia, Intracranial-extracranial tumors, Neurosurgery, Oral-maxillofacial surgery, Perioperative, Skull base tumors

## Abstract

**Background:**

To analyze the clinical characteristics of patients with complex skull base lesions involving both intracranial and extracranial regions and summarize the anesthetic management experience for combined neurosurgical and oral-maxillofacial procedures under a single anesthesia session.

**Case presentation:**

Medical records of patients who underwent combined neurosurgical and oral-maxillofacial surgery at Beijing Tiantan Hospital, Capital Medical University, between April 1, 2020, and April 1, 2025, were retrospectively reviewed. Clinical data, multidisciplinary treatment, anesthetic management, and outcomes were analyzed. Six patients underwent multidisciplinary consultation before surgery, using a combination of static and inhalation anesthesia. Three (50%) cases were intubated through nasal endotracheal intubation, two (33.3%) cases were intubated through tracheal intubation, and one (16.7%) case was intubated through tracheostomy after anesthesia. All 6 patients were admitted to the ICU with endotracheal intubation or tracheostomy tube after surgery. 5 (83.3%) patients recovered well and were discharged, while 1 (16.7%) patient died.

**Conclusion:**

Complex skull base lesions with intracranial and extracranial involvement are rare but may lead to severe complications if not promptly managed [1]. Multidisciplinary collaboration among neurosurgery, oral-maxillofacial surgery, anesthesiology, and neurophysiology teams is critical. Anesthesia management should not only be limited to intraoperative procedures, but should also pay attention to the entire perioperative period, especially the timing of patient extubation.

**Supplementary Information:**

The online version contains supplementary material available at 10.1186/s12871-025-03310-9.

## Background

Intracranial-extracranial communicating tumors represent a distinct category of neoplasms that originate either intracranially or extracranially, infiltrate the skull base during growth, and eventually form lesions spanning both compartments [1, 2]. These tumors often occupy anatomically complex regions that may lead to communications, as schwannoma [3–6]. Due to their indolent progression and lack of early cranial nerve compression, some patients remain asymptomatic until the tumor reaches an advanced stage, occasionally presenting with facial deformities. The medical record of complete tumor resection and skull base reconstruction in one surgery is very rare, and perioperative management is difficult. Currently, no standardized treatment protocol exists, warranting further exploration. This study summarizes case data from combined neurosurgical and oral-maxillofacial procedures at Beijing Tiantan Hospital, Capital Medical University, and reviews the literature to identify key perioperative management strategies.

## Case presentation

This study was approved by the Ethics Committee of Beijing Tiantan Hospital, Capital Medical University (KY 2019-124-02), date of approval (19/11/2019). We retrospectively analyzed medical records of patients who underwent combined neurosurgical and oral-maxillofacial surgery between April 1, 2020, and April 1, 2025.

### Demographic characteristics

Among the 6 patients, 4 (66.7%) were male with a mean age of 29 ± 7.9 years. Three patients (50%) presented with tumor recurrence, including one case of severe anemia (hemoglobin 5.1 g/dL) due to impaired wound healing following postoperative chemoradiotherapy for melanoma. Two patients (33.3%) exhibited sinus tachycardia upon admission, while one (16.7%) demonstrated complete right bundle branch block. Preoperative MRI confirmed hypervascular tumors in all cases (Table 1).

**Table 1:** Patient demographics and baseline data

No.	Age (years)	Sex	Medical history	Weight (kg)	Height (cm)	BMI (kg/m²)	Tumor location	Tumor size (L × W × H, cm)	Clinical symptoms
1	36	M	Severe anemia, sinus tachycardia, status post melanoma resection (with poor wound healing)	75	176	24.2	Right occipital region and neck	5.6 × 4.2 × 6.6	Neck weakness, pain in the right shoulder, and difficulty moving the right upper limb for 2 years
2	28	M	Sinus tachycardia, status post resection of right orbital adenoid cystic carcinoma	65	180	20.1	Right orbit, right ethmoid sinus, and right frontal lobe	5.5 × 5.5 × 9.0	Discomfort in the right eye and a one-year decline in vision
3	34	M	N	65	174	21.5	Left frontotemporal region, left intraorbital space, sphenoid sinus, ethmoid sinus, and parasellar region	11.5 × 7.7 × 9.4	The left frontal and temporal regions gradually protrude for 8 years, while the left eyeball protrudes, vision declines, and epilepsy persists for 1 year
4	14	M	N	75	178	23.7	Left middle cranial fossa, infratemporal fossa, bilateral sphenoid sinuses, left maxillary sinus, pterygopalatine fossa, and nasopharynx	6.8 × 7.5 × 10.4	Bilateral facial asymmetry for 3 years
5	29	F	CRBBB	65	153	27.8	Left mastoid cavity and left cerebellopontine angle	6.6 × 4.1 × 3.3	Left ear hearing loss for 2 years
6	33	F	Postoperative status of chordoma resection	50	165	18.4	Right posterior cranial fossa, clivus, peri-C1-C2 vertebral region, nasopharyngeal cavity, spinal canal, and right petroclival region	6.7 × 5.0 × 5.5	Headache and hoarseness for 1 year, poor breathing for 2 months

*M *male, *F* Female, *CRBBB* Complete right bundle branch block, *Y* Yes, *N* No, *BMI*: Body mass index (calculated as weight in kilograms divided by height in meters squared)

### Intraoperative management

ASA physical status distribution was: 3 cases (50%) class II, 1 (16.7%) class III, and 2 (33.3%) class IV. Six patients were all anesthetized with a combination of intravenous and inhalation anesthesia, with 3 (50%) undergoing nasotracheal intubation, 2 (33.3%) orotracheal intubation, and 1 (16.7%) post-induction tracheostomy. Vasopressor support included norepinephrine in 4 cases (66.7%) and phenylephrine in 2 (33.3%). Median surgical duration was 11 (10–12) hours with estimated blood loss of 1750 (800–2700) mL (Table 2).

**Table 2:** Procedural characteristics and intraoperative performance

No.	ASA grade	Anesthesia method	Intubation site	Baseline BP(mmHg)	Post-induction BP(mmHg)	Lowest intraop BP(mmHg)	Duration of hemodynamic instability(min)	Total operative time(h)	Anesthesia duration(h)	Vasoactive drugs	Duration of vasoactive drug infusion(h)	Urine output(ml)	Blood loss(ml)	Crystalloid(ml)	Colloid(ml)	Total fluids(ml)	Blood transfusion
1	IV	ii	O	160/110	120/70	70/50	30	12	13	PHE	11	2200	400	4000	1000	5000	RBCs 4U + P 400 ml
2	III	ii	N	120/80	100/70	80/50	60	10	12	NA	8	2800	2700	7000	1000	8000	RBCs 6U + P 400 ml
3	II	ii	O	120/70	100/60	70/50	120	15	19	NA	7	5800	5600	8500	2000	10,500	ABT 1000 ml + RBCs 10U + P 1600 ml
4	II	ii	N	160/70	110/60	70/50	120	12	13	NA	7	2300	2700	4700	1000	5700	ABT 500 ml + RBCs 4U + P 800 ml
5	II	ii	N	110/70	90/50	80/50	30	10	11.5	NA	6	1200	800	3500	500	4000	RBCs 2U + P 400 ml
6	IV	ii	T	110/70	90/50	80/40	30	6.5	7.5	PHE	6	1000	800	2100	500	2600	RBCs 2U + P 400 ml

ii Combined intravenous-inhalational anesthesia, *O* Orotracheal intubation, *N* Nasotracheal intubation, *T* Tracheotomy tube placement, *PHE* Phenylephrine, *NA* Norepinephrine, *ABT* Autologous blood transfusion, *RBCs* Red blood cells, *P* Plasma

### Postoperative outcomes

All 6 patients were admitted to the ICU with endotracheal intubation or tracheostomy tube after surgery. 4 (66.7%) patients recovered spontaneous breathing and left the operating room in a drowsy state. 2 (33.3%) patients did not recover spontaneous breathing and left the operating room under anesthesia. Five patients with endotracheal intubation had their tubes removed within 2 (2,5) days. 1 (16.7%) tracheostomy patients were discharged with tracheostomy tubes. 1 (16.7%) patients experienced worsening of their condition after surgery, with sudden respiratory distress and slowed heart rate 14 days after surgery. Immediate cardiopulmonary resuscitation and intubation were administered, but they died due to ineffective rescue efforts. The remaining 5 (83.3%) patients recovered well and were discharged (Table 3).

**Table 3:** Postoperative clinical characteristics and outcomes

No.	Surgical procedure	Extubation in OR	Consciousness recovery	Spontaneous breathing	Complications	Time to extubation (days)	Hospital stay (days)	Pathology	Prognosis
1	Occipitocervical mass resection + Latissimus dorsi myocutaneous flap reconstruction	N	N	N	Anemia and weakened cough strength	1	21	Melanoma	Sudden limb movement sensation disorder occurred 7 days after surgery. Sudden respiratory distress and slowed heart rate 14 days after surgery. Death due to ineffective rescue efforts.
2	Right fronto-orbito-zygomatic approach for ethmoid/maxillary sinus tumor resection with orbital exenteration + skull base reconstruction + pedicled latissimus dorsi flap transfer	N	N	Y	N	2	12	Adenoid cystic carcinoma	Recovered well and discharged from the hospital.
3	Left frontotemporal craniotomy for intracranial lesion resection + cranioplasty + left eyelid canthoplasty	N	N	N	Anemia and hypoalbuminemia	2	13	Transitional meningioma	Recovered well and discharged from the hospital.
4	Left frontotemporal zygomatic osteotomy for infratemporal lesion resection	N	N	Y	N	6	17	Schwannoma	Recovered well and discharged from the hospital.
5	Retroauricular curvilinear craniotomy for tumor resection + left subaxillary muscle-fascia flap harvest + left skull base defect repair with pedicled flap reconstruction	N	N	Y	Coma, agitation, and pulmonary infection	5	15	Cholesteatoma	Recovered well and discharged from the hospital.
6	Transoral approach for clival tumor resection	N	N	Y	N	N	13	Chordoma	Discharged with tracheostomy tube.

*Y* Yes, *N* No

### Statistical analysis

Data were analyzed using SPSS 24.0. Normally distributed continuous variables are expressed as mean ± standard deviation ($\:\stackrel{\prime }{x}$± s); non-normal data as median [interquartile range] [M (Q1, Q3)]; and categorical variables as counts (percentages).

## Discussion

Intracranial-extracranial communicating tumors can affect structures and functions in multiple regions including brain tissue, facial structures, skull base, and neck, leading to various symptoms such as headache, visual impairment, facial numbness, and hearing loss [1, 2]. These rare tumors demonstrate diverse pathological types, with malignant variants being predominant [7–13]. The complex regional anatomy with numerous neurovascular structures poses significant surgical challenges. Completing a combined neurosurgery and oral and maxillofacial surgery under anesthesia poses a significant challenge for the surgical team. We have reached the following conclusions in this medical record summary, hoping to provide some guidance for perioperative management of such patients in the future.

### Multidisciplinary preoperative evaluation

A multidisciplinary joint consultation should be conducted before surgery to prepare adequately for the procedure. Neurosurgery, oral and maxillofacial surgery, and anesthesia departments should jointly conduct preoperative discussions and develop surgical plans. Emphasize the evaluation of the patient’s tumor size, location, surrounding neurovascular conditions, and blood supply. Explore possible emergency situations during surgery (such as nerve damage; tumor invasion of large blood vessels and nerves; abundant tumor blood supply; circulation fluctuations caused by nerve traction; whether to perform skin flap transplantation for skull base reconstruction; site and survival rate of skin flap transplantation, etc.) and corresponding measures [14–18].

### Anesthesia management challenges

Preoperative evaluation of 6 patients revealed tumor invasion of surrounding tissues, with a large volume and abundant blood supply. Therefore, emergency plans for anesthesia management of major bleeding were prepared before surgery [19–21]. After general anesthesia, promptly open the central venous catheter and prepare an autologous blood recovery device to recover the patient’s craniotomy blood (blood before starting to remove malignant tumors). Open invasive arterial pressure monitoring to monitor the patient’s circulatory status in real-time. Actively replenishing fluids and using vasoactive drugs for symptomatic supportive treatment during surgery. Avoiding the passive situation of excessive bleeding during the surgery, timely monitoring of blood gas conditions during the operation. The first patient had severe anemia before operation, but the operation was needed as soon as possible due to the serious condition and poor wound healing. Therefore, special attention should be paid to contacting the transfusion department for blood collection before the surgery begins, and the surgery should be performed after correcting anemia. The third patient had the largest tumor volume, and during craniotomy, it was found that the tumor had invaded and adhered to the venous sinus, resulting in unpredictable massive bleeding. The instantaneous bleeding volume was 2000 ml, causing significant fluctuations in circulation. The instantaneous blood pressure was 70/50mmHg, and the heart rate was 140 beats per minute. Timely replace the experienced lead surgeon to compress and suture the bleeding, and immediately administer autologous blood for craniotomy, as well as vascular active drugs and fluid replacement for symptomatic treatment. Timely infusion of allogeneic blood during subsequent tumor resection to maintain stable intraoperative circulation, gradually discontinuing vasoactive drugs.

### Intraoperative neuromonitoring

During neurosurgery, attention should be paid to the protection of neurological function to avoid postoperative motor or sensory dysfunction caused by surgical injuries [22–24]. This study used intraoperative electrophysiological monitoring to monitor the patient’s upper and lower limb sensation, movement, and cranial nerve function in real-time. To ensure monitoring accuracy, anesthesia is maintained using a combination of intravenous and inhalation anesthesia (with an MCA controlled below 0.5), and no muscle relaxants are added during monitoring. In this study, 6 patients had good neurological function protection during surgery and no symptoms of nerve damage occurred after surgery.

### Postoperative considerations

Patients undergoing large-scale skull base reconstruction surgery, especially those undergoing skin flap transplantation, should pay attention to the survival of the skin flap after surgery, observe the wound in a timely manner, and avoid complications such as infection [14–18]. Previous studies have shown that endoscopic sinus surgery is already mature for tumors in the sellar region. For intracranial and extracranial communicating lesions in this area, attention should be paid to reconstructing the skull base with the nasal septal flap, in order to effectively prevent postoperative cerebrospinal fluid leakage [25, 26]. All 6 patients received prophylactic treatment with anti infective drugs after surgery. At the same time, due to the large size of the tumor, excessive bleeding, long surgical time, and significant intracranial edema, hormone anti edema treatment was adopted after surgery. For patients with high intracranial pressure, mannitol dehydration is given to reduce intracranial pressure, and postoperative monitoring of patient intake and output, hemoglobin, albumin, and electrolyte levels is conducted. The focus of postoperative management should be on correcting anemia and hypoalbuminemia, ensuring the blood supply and nutrition of the transplanted skin flap, and guaranteeing its survival. And in terms of airway management and lung protection for patients with retained tracheal intubation (tracheostomy tube).

### Airway management

Anesthesia management should not only focus on fluid management and water electrolyte acid-base balance during intraoperative bleeding, but also extend to the perioperative period, especially in controlling the timing of extubation. Giant intracranial and extracranial communication tumors, due to their invasiveness, often cause damage to the skull and surrounding tissues, with a wide range of involvement. Once the tumor invades the cervical canal and surrounding tissues, it is easy to cause high cervical spinal cord nerve injury in patients. Postoperative complications such as sudden respiratory distress and slowed heart rate are prone to occur. In this study, all 6 patients retained tracheal intubation or tracheostomy tube after surgery to ensure ventilation. However, the first patient failed to detect tumor invasion into the foramen magnum and atlantoaxial vertebrae in a timely manner, resulting in cervical bone destruction and poor cervical stability. Postoperative failure to use cervical brace to protect cervical spine and premature removal of endotracheal tube. Although timely detection of changes in the patient’s condition and timely implementation of re intubation and cardiopulmonary resuscitation rescue were carried out, serious consequences still occurred. This kind of situation makes us more vigilant and avoid related risks in the future diagnosis and treatment process.

### Postoperative anesthesia complications

Due to the extensive surgical trauma, prolonged operative duration, and significant intraoperative blood loss, special vigilance for anesthesia-related complications is warranted postoperatively. Close monitoring of body temperature in patients with major hemorrhage is essential to prevent complications such as shivering induced by hypothermia. Additionally, meticulous attention should be paid to fluid-electrolyte and acid-base imbalances, with timely arterial blood gas analysis and active symptomatic treatment. For patients requiring retained endotracheal intubation, appropriate sedation during ICU transfer is crucial to prevent postoperative delirium and agitation.

### Study limitations

The small sample size (*n* = 6) and single-center design limit generalizability. Future studies will involve continued collection of relevant cases and attempts to conduct multicenter prospective research to further explore the key points of perioperative management for this category of diseases.

## Conclusion

The present study advocates that patients with complex skull base tumors involving both intracranial and extracranial compartments should undergo multidisciplinary team (MDT) consultation preoperatively. Intraoperative management should prioritize neural function preservation and massive hemorrhage control, while postoperative care necessitates rigorous vital sign monitoring to optimize extubation timing. With adequate preparation and collaborative multidisciplinary efforts, these patients can achieve improved clinical outcomes.

## Supplementary Information

Supplementary Material 1.

## Data Availability

No datasets were generated or analysed during the current study.
