# Supplementary material for: Perioperative management of intracranial-extracranial communicating tumors with multidisciplinary combined surgery: a case series
Source: BMC Anesthesiol. 2025 Aug 27;25:427. doi: 10.1186/s12871-025-03310-9 (PMC12382093; doi:10.1186/s12871-025-03310-9)
Supplement: Supplementary file 1 — Supplementary Material 1. [file 12871_2025_3310_MOESM1_ESM.docx]

**Table 1. Patient demographics and baseline data**

| **No.** | **Age (years)** | **Sex** | **Medical history** | **Weight (kg)** | **Height (cm)** | **BMI (kg/m²)** | **Tumor location** | **Tumor size (L × W × H, cm)** | **Clinical symptoms** |
| --- | --- | --- | --- | --- | --- | --- | --- | --- | --- |
| 1 | 36 | M | **Severe anemia, sinus tachycardia, status post melanoma resection (with poor wound healing)** | 75 | 176 | 24.2 | **Right occipital region and neck** | 5.6×4.2×6.6 | Neck weakness, pain in the right shoulder, and difficulty moving the right upper limb for 2 years |
| 2 | 28 | M | **Sinus tachycardia, status post resection of right orbital adenoid cystic carcinoma** | 65 | 180 | 20.1 | **Right orbit, right ethmoid sinus, and right frontal lobe** | 5.5×5.5×9.0 | Discomfort in the right eye and a one-year decline in vision |
| 3 | 34 | M | N | 65 | 174 | 21.5 | **Left frontotemporal region, left intraorbital space, sphenoid sinus, ethmoid sinus, and parasellar region** | 11.5×7.7×9.4 | The left frontal and temporal regions gradually protrude for 8 years, while the left eyeball protrudes, vision declines, and epilepsy persists for 1 year |
| 4 | 14 | M | N | 75 | 178 | 23.7 | **Left middle cranial fossa, infratemporal fossa, bilateral sphenoid sinuses, left maxillary sinus, pterygopalatine fossa, and nasopharynx** | 6.8×7.5×10.4 | Bilateral facial asymmetry for 3 years |
| 5 | 29 | F | CRBBB | 65 | 153 | 27.8 | **Left mastoid cavity and left cerebellopontine angle** | 6.6×4.1×3.3 | Left ear hearing loss for 2 years |
| 6 | 33 | F | Postoperative status of chordoma resection | 50 | 165 | 18.4 | **Right posterior cranial fossa, clivus, peri-C1-C2 vertebral region, nasopharyngeal cavity, spinal canal, and right petroclival region** | 6.7×5.0×5.5 | Headache and hoarseness for 1 year, poor breathing for 2 months |

**Notes:** M: male; F: female; CRBBB: complete right bundle branch block; Y: yes; N: no; BMI: body mass index (calculated as weight in kilograms divided by height in meters squared).

**Table 2. Procedural characteristics and intraoperative performance**

| No. | **ASA grade** | **Anesthesia method** | **Intubation site** | **Baseline BP**（mmHg） | **Post-induction BP**（mmHg） | **Lowest intraop BP**（mmHg） | Duration of hemodynamic instability（min） | Total operative time（h） | Anesthesia duration（h） | Vasoactive drugs | **Duration of vasoactive drug infusion**（h） | **Urine output**（ml） | **Blood loss**（ml） | **Crystalloid**（ml） | **Colloid**（ml） | **Total fluids**（ml） | **Blood transfusion** |
| --- | --- | --- | --- | --- | --- | --- | --- | --- | --- | --- | --- | --- | --- | --- | --- | --- | --- |
| 1 | Ⅳ | ii | O | 160/110 | 120/70 | 70/50 | 30 | 12 | 13 | PHE | 11 | 2200 | 400 | 4000 | 1000 | 5000 | RBCs 4U+P 400ml |
| 2 | Ⅲ | ii | N | 120/80 | 100/70 | 80/50 | 60 | 10 | 12 | NA | 8 | 2800 | 2700 | 7000 | 1000 | 8000 | RBCs 6U+P 400ml |
| 3 | Ⅱ | ii | O | 120/70 | 100/60 | 70/50 | 120 | 15 | 19 | NA | 7 | 5800 | 5600 | 8500 | 2000 | 10500 | ABT 1000ml+RBCs 10U+P 1600ml |
| 4 | Ⅱ | ii | N | 160/70 | 110/60 | 70/50 | 120 | 12 | 13 | NA | 7 | 2300 | 2700 | 4700 | 1000 | 5700 | ABT 500ml+RBCs 4U+P 800ml |
| 5 | Ⅱ | ii | N | 110/70 | 90/50 | 80/50 | 30 | 10 | 11.5 | NA | 6 | 1200 | 800 | 3500 | 500 | 4000 | RBCs 2U +P 400ml |
| 6 | Ⅳ | ii | T | 110/70 | 90/50 | 80/40 | 30 | 6.5 | 7.5 | PHE | 6 | 1000 | 800 | 2100 | 500 | 2600 | RBCs 2U+P 400ml |

Notes：ii：**Combined intravenous-inhalational anesthesia**；O：**Orotracheal intubation**；N：**Nasotracheal intubation**；T：**Tracheotomy tube placement**；PHE：**Phenylephrine**；NA：**Norepinephrine; ABT: Autologous blood transfusion; RBCs: Red blood cells; P: Plasma**

**Table 3. Postoperative clinical characteristics and outcomes**

| **No.** | **Surgical procedure** | **Extubation in OR** | **Consciousness recovery** | **Spontaneous breathing** | **Complications** | **Time to extubation** (days) | **Hospital stay** (days) | **Pathology** | **Prognosis** |
| --- | --- | --- | --- | --- | --- | --- | --- | --- | --- |
| 1 | **Occipitocervical mass resection + Latissimus dorsi myocutaneous flap reconstruction** | N | N | N | Anemia and weakened cough strength | 1 | 21 | **Melanoma** | Sudden limb movement sensation disorder occurred 7 days after surgery. Sudden respiratory distress and slowed heart rate 14 days after surgery. Death due to ineffective rescue efforts. |
| 2 | **Right fronto-orbito-zygomatic approach for ethmoid/maxillary sinus tumor resection with orbital exenteration + skull base reconstruction + pedicled latissimus dorsi flap transfer** | N | N | Y | N | 2 | 12 | **Adenoid cystic carcinoma** | Recovered well and discharged from the hospital. |
| 3 | **Left frontotemporal craniotomy for intracranial lesion resection + cranioplasty + left eyelid canthoplasty** | N | N | N | **Anemia and hypoalbuminemia** | 2 | 13 | **Transitional meningioma** | Recovered well and discharged from the hospital. |
| 4 | **Left frontotemporal zygomatic osteotomy for infratemporal lesion resection** | N | N | Y | N | 6 | 17 | **Schwannoma** | Recovered well and discharged from the hospital. |
| 5 | **Retroauricular curvilinear craniotomy for tumor resection + left subaxillary muscle-fascia flap harvest + left skull base defect repair with pedicled flap reconstruction** | N | N | Y | **Coma, agitation, and pulmonary infection** | 5 | 15 | Cholesteatoma | Recovered well and discharged from the hospital. |
| 6 | **Transoral approach for clival tumor resection** | N | N | Y | N | N | 13 | **Chordoma** | Discharged with tracheostomy tube. |

Note: Y: Yes; N: No;
